# Developing Culturally Competent Technology for Older Adults in Japan and the United States

**DOI:** 10.1093/geroni/igab046.1747

**Published:** 2021-12-17

**Authors:** Laura Allen, Dana Bradley, Gretchen Tucker

**Affiliations:** 1 Bar-Ilan University, Ramat Gat, Tel Aviv, Israel; 2 UMBC Erickson School of Aging Studies, Baltimore, Maryland, United States

## Abstract

The United States and Japan are experiencing an exponential growth in the number of persons age 65 and older. To address certain aging-related issues, assistive technological advancements are being developed. These technologies need to be reliable, safe, secure, and culturally accepted by older adults. In addition, technology must be developed within the unique cultural contexts of each country. One approach currently being used is an interdisciplinary team approach comprised of researchers representing gerontology, information systems, robotics, health sciences, sociology, and computer sciences between two universities in the United States and two Japanese universities. This collaborative project between institutions and countries highlights the need to understand the cultures and traditions of each of these countries. To further develop culturally competent technology, an integrative research plan is being utilized, which incorporates the use of community engagement to examine the influence of the cultural context among older adults.

